# 232 Enhance active ageing by promoting appropriate lifestyles through the endorsement of healthy behaviours in an ageing Region

**DOI:** 10.1093/eurpub/ckae114.132

**Published:** 2024-09-26

**Authors:** Laura Pagani, Tiziana Del Fabbro, Luana Sandrin, Gianna Zamaro, Alessia Del Bianco Rizzardo

**Affiliations:** Department Of Economics And Statistics - University Of Udine, Udine, Italy; Federsanità ANCI FVG, Udine, Italy; Health Directorate, Autonomous Region Friuli Venezia Giulia, Italy, Trieste, Italy; Health Directorate, Autonomous Region Friuli Venezia Giulia, Italy, Trieste, Italy; PromoTurismoFVG, Accessible and Sustainable Tourism, Autonomous Region Friuli Venezia Giulia, Italy, Trieste, Italy

## Abstract

**Purpose:**

The project “Friuli Venezia Giulia in Movimento, 10000 passi di salute” (Friuli Venezia Giulia (FVG) Region on the move, 10000 healthy steps) commenced in January 2019 with financial support from the FVG Region with the organizational support of a regional association (Federsanità ANCI FVG). The project’s primary objective is to engage municipalities, in collaboration with organized and spontaneous associations, in implementing initiatives to promote physical activity in an accessible manner for all citizens. This is achieved through funding to promote pedestrian paths in municipalities that have chosen to participate, as well as walking leader courses and gentle exercise activities for the elderly. The project involves subjects at different governance levels: region, the regional association, Municipalities, citizens’ associations, and citizens.

**Project or policy description:**

Currently, 91 municipalities (42% of the FVG Region) are participating in the project, with 82 pedestrian paths covering a total length of over 400 km now open. Specifically, 52% of individuals over 65 and 56% of those under 15 will benefit from the activities organized along these paths. Additionally, two editions of walking leader courses have been conducted along the project’s pedestrian paths, with 500 participants, and two editions of gentle exercise activities for the elderly have been held in 19 municipalities, involving over 1150 individuals. Many Municipalities have also organized walks along the project’s pedestrian paths, often accompanied by cultural events. Traditional and social media channels have prioritised communication and dissemination of project initiatives. Furthermore, numerous citizen meetings have been convened to introduce the projects and related initiatives. The project has been continuously evaluated, with activity results documented in reports. The evaluation involves different phases and all the actors that the project is intended for with various tools: it is, therefore, a multilevel evaluation.

**Conclusions:**

The project has significantly impacted promoting active ageing through various activities, including physical exercise, social engagement, well-being enhancement, and local area awareness. Given their conformation (flat and without particular obstacles), the regional tourism agency has promoted the pedestrian paths of the project within the context of slow, sustainable, and inclusive tourism.

